# Supplementary material for: Altered increase in STAT1 expression and phosphorylation in severe COVID‐19
Source: Eur J Immunol. 2021 Nov 17;52(1):138–48. doi: 10.1002/eji.202149575 (PMC8646801; doi:10.1002/eji.202149575)
Supplement: Supplementary file 1 — Figure S1 Reduced STAT2 expression in severe COVID‐19 patients. (A) Median fluorescence intensity (MFI) of STAT2 in CD3+, CD19 and CD14+ cells, as well in T and B cell subsets. Median from healthy controls (n = 3), mild COVID‐19 (n = 7) and severe COVID‐19 (n = 5) patients. (B) MFI of pSTAT2 in in CD3+, CD19 and CD14+ cells, as well in T and B cell subsets. Median from healthy controls (n = 20), mild COVID‐19 (n = 17) and severe COVID‐19 (n = 13) patients. (C) Ratio pSTAT/STAT1 in CD3+, CD19 and CD14+ cells, as well in T and B cell subsets. Median from healthy controls (n = 20), mild COVID‐19 (n = 17) and severe COVID‐19 (n = 13) patients. Two way ANOVA with Sidack post‐test. *p<0.05, **p<0.01, ***p<0.001 and ****p<0.0001. Deceased patients are indicated as purple quadrats. [file EJI-52-138-s001.pdf]

Fig S1

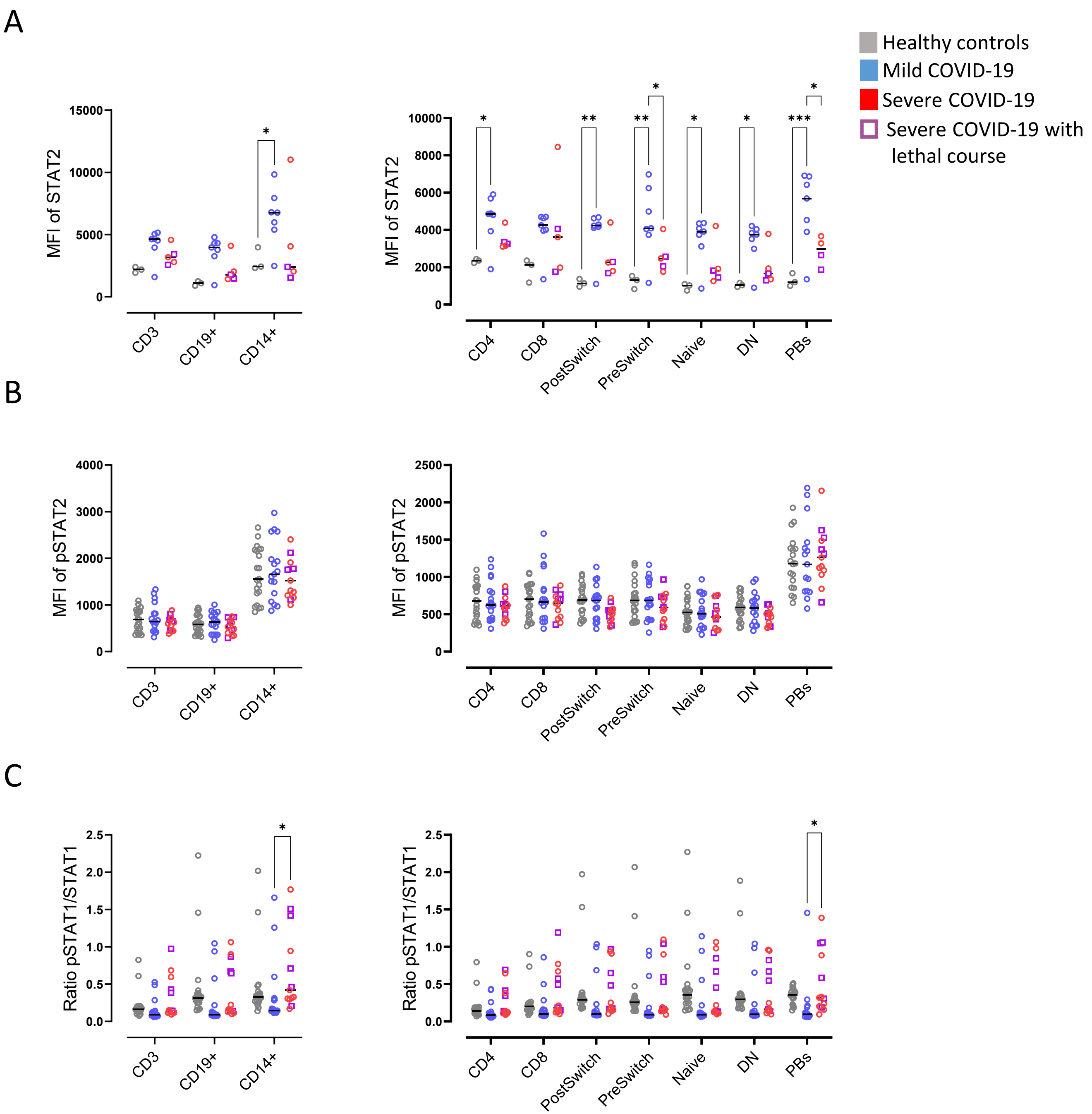
A
B
C
Healthy controls
Mild COVID-19
Severe COVID-19
Severe COVID-19 with lethal course
MFI of STAT2
MFI of pSTAT2
Ratio pSTAT1/STAT1
CD3
CD19+
CD14+
CD4
CD8
PostSwitch
PreSwitch
Naive
DN
PBs
*
**
***

Figure S1 Reduced STAT2 expression in severe COVID-19 patients

(A) Median fluorescence intensity (MFI) of STAT2 in CD3+, CD19 and CD14+ cells, as well in T and B cell subsets. Median from healthy controls (n=3), mild COVID-19 (n=7) and severe COVID-19 (n=5) patients. (B) MFI of pSTAT2 in in CD3+, CD19 and CD14+ cells, as well in T and B cell subsets. Median from healthy controls (n=20), mild COVID-19 (n=17) and severe COVID-19 (n=13) patients. (C) Ratio pSTAT/STAT1 in CD3+, CD19 and CD14+ cells, as well in T and B cell subsets. Median from healthy controls (n=20), mild COVID-19 (n=17) and severe COVID-19 (n=13) patients. Two way ANOVA with Sidack post-test. *p<0.05, **p<0.01, ***p<0.001 and ****p<0.0001**.** Deceased patients are indicated as purple quadrats.
